# Effortful Control and Cortical Brain Structure in 5‐Year‐Old Children: Findings From the FinnBrain Birth Cohort Study

**DOI:** 10.1111/ejn.70580

**Published:** 2026-06-10

**Authors:** Meri Frantti, Jetro J. Tuulari, Saara Nolvi, Elisabeth Nordenswan, Anni Copeland, Venla Kumpulainen, Eero Silver, Harri Merisaari, Ekaterina Saukko, Eeva‐Leena Kataja, Riikka Korja, Linnea Karlsson, Hasse Karlsson, Elmo P. Pulli

**Affiliations:** ^1^ FinnBrain Birth Cohort Study, Turku Brain and Mind Center, Department of Clinical Medicine University of Turku Turku Finland; ^2^ Centre for Population Health Research Turku University Hospital and University of Turku Turku Finland; ^3^ Clinical Neurosciences, Faculty of Medicine University of Turku Turku Finland; ^4^ Neurocenter Turku University Hospital Turku Finland; ^5^ Department of Psychology and Speech‐Language Pathology University of Turku Turku Finland; ^6^ The Centre of Excellence for Learning Dynamics and Intervention Research (InterLearn) University of Turku and University of Jyväskylä Jyväskylä Finland; ^7^ Department of Radiology Turku University Hospital and University of Turku Turku Finland; ^8^ Department of Public Health University of Turku and Turku University Hospital Turku Finland; ^9^ Department of Child Psychiatry Turku University Hospital Turku Finland; ^10^ Department of Clinical Medicine, Psychiatry University of Turku and Turku University Hospital Turku Finland

**Keywords:** effortful control, MRI, neuroimaging, self‐regulation, temperament

## Abstract

The aim of this study was to explore the associations between an aspect of self‐regulation (SR), effortful control (EC) and cortical brain structure in 5‐year‐old children. Efficient EC is a predictor of many attributes and important outcomes in life, such as social–emotional functioning, finance, psychiatric and somatic health. The early brain correlates of EC are not widely studied, and a better understanding of them would aid in understanding how self‐regulatory capacities emerge over development. Participants (*N* = 155) were a part of the FinnBrain Birth Cohort Study in Finland. T1‐weighted brain magnetic resonance images were processed using FreeSurfer. The data were statistically analysed with a vertex‐wise general linear model. At the age of 5 years, EC was assessed via parental report using The Children's Behaviour Questionnaire. We found positive associations between EC and cortical volume in the left supramarginal region and in the right inferior temporal region. We also found positive associations between EC and surface area on the left hemisphere in the superior parietal region. We extended the previous literature by shedding light on early structural brain correlates of EC in a large sample of typically developing 5‐year‐olds. The main results differed significantly from previous findings in older children. The results were only present with questionnaire‐ and not task‐based evaluation of EC. Both questionnaire and task‐based evaluations are required to consider different aspects of EC and SR. In addition, longitudinal studies are needed to better understand the neural underpinnings of SR throughout development.

## Graphical abstract

The structural correlates of effortful control in typically developing 5‐year‐olds were found in the left supramarginal/superior parietal region and in the right inferior temporal region. The results differed from those seen in older participants in earlier studies. The novelty of this study is in its large sample size within a narrowly defined age range.

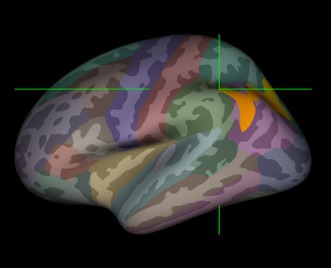

**Abbreviations**
ACC: anterior cingulate cortex
ATTFO: attentional focusing
BMI: body mass index
CBCL: Child Behaviour Checklist
CBQ: Children's Behaviour Questionnaire
EC: effortful control
EPDS: Edinburgh Postnatal Depression Scale
GM: grey matter
INH: inhibitory control
LIP: low intensity pleasure
MRI: magnetic resonance imaging
PER: perceptual sensitivity
PFC: prefrontal cortex
SCL‐90: Symptom Check List 90
SD: standard deviation
SR: self‐regulation

## 1 Introduction

Self‐regulation (SR) refers to the capacity to modify one's behaviour, emotion and cognition in accordance with the environmental framework (Cole et al. 2019; Nigg 2017). It affects actions and emotions through contributing to the ability to recognize, express and suppress them in a socially adaptive manner. SR can also be considered a part of personality that consists of innate characteristics and is shaped by the experiences in life. It is an umbrella concept that includes both temperamental and cognitive regulatory processes, such as executive function, effortful control (EC) and emotion regulation (Bridgett et al. 2015; Nigg 2017).

In this study, we chose to approach SR through temperament traits (Goldsmith et al. 1987). Rothbart's psychobiological theory of temperament conceptualizes temperament in young children as the individual differences in reactivity and regulation, with reactivity divided into positive and negative emotional reactivity, and aspects of regulation usually referred to as EC (Rothbart 2007). In this study, we focus on EC, which represents the ability to control one's actions under conflict, plan future actions and detect errors (Rothbart 2007; Nigg 2017). Thus, EC can be considered a trait‐level aspect of top‐down SR (Nigg 2017). The precursors of EC are observed already in infancy (Eisenberg et al. 2014), but moderate stability of EC is reached from toddlerhood or preschool onwards (Rothbart 2007). Childhood EC also predicts adulthood conscientiousness, a personality trait linked with SR and favourable life outcomes (Eisenberg et al. 2014; Pérez‐Edgar and Fox 2005; Rothbart 2007).

EC and other aspects of SR are important predictors for a myriad of later life outcomes: Higher self‐control in childhood is positively linked to social skills and academic achievement and negatively linked to aggressive and criminal behaviour, mental and physical health issues, obesity and cigarette smoking (Robson et al. 2020). Lower EC during adolescence is also linked to the risk of subsequent substance use disorder (Cheetham et al. 2017; Robson et al. 2020). In addition, higher EC at 2–8 years was associated with more prosocial behaviour and less internalizing and externalizing symptoms (Robson et al. 2020; Slobodskaya et al. 2020). A better understanding of the early underlying mechanisms of EC could help identify developmental models of SR and provide a foundation for hypotheses regarding later developmental pathways.

Particularly, the knowledge of early neurobiological factors underlying EC could be of interest in understanding the dynamic development of SR and EC. Traditionally, as reviewed by Kelley et al. (2015) and Fiske and Holmboe (2019), the main neural areas linked to executive function, a concept which includes inhibitory control (INH), working memory and cognitive shifting, are thought to be the prefrontal cortex (PFC), particularly the ventromedial and the lateral regions and the anterior cingulate cortex (ACC). Currently, there are few child or adolescent magnetic resonance imaging (MRI) studies that used questionnaire data investigating SR in real‐life contexts (see Supporting Information S1). Out of the 20 studies found, only two included both MRI and SR assessment questionnaire of children under 6 years of age. Notably, the neuroanatomical findings in studies with younger participants differ from those usually seen in older participants (Kelley et al. 2015). Some studies were on special populations, for example, Hadaya et al. (2023) conducted a study where children born preterm had an MRI scan at 38–53 weeks post‐menstrual age and a neuropsychological follow‐up at 4–7 years. Interestingly, the subgroup that had the highest EC scores also displayed larger relative volumes in the left insula and bilateral orbito‐frontal cortex (OFC) and higher degree centrality in an overlapping region in the left OFC, suggesting a link between structural variation in these anatomical areas and self‐regulatory capacities. However, with the imaging and neuropsychological evaluation being years apart, correlation between neuroanatomical structures and behaviour can only be speculated. In addition, research on special populations such as preterm‐born children should be complemented by research on general population participants.

To summarize, the research on early childhood neural underpinnings of EC, an aspect of SR, remains relatively limited, particularly in typically developing preschool‐aged populations, and especially in studies using questionnaires. Questionnaire data may capture more of the everyday context of SR in comparison with the neuropsychological assessment tasks performed in a laboratory setting, which typically assess more specific and narrow aspects of SR. To the best of our knowledge, this is the first large cross‐sectional study examining EC and brain structure in preschool‐age general population children.

Some earlier studies have examined this topic in a sample with a wider age range (Badaly et al. 2022; Feng et al. 2023). As both the brain and EC are still in a developing state at 5 years of age, the neural basis of EC could be substantially different from that in older participants (Hadaya et al. 2023). Furthermore, grey matter (GM) volume reaches its peak at 6 years of age (Bethlehem et al. 2022; Courchesne et al. 2000) and then starts to decrease (Bethlehem et al. 2022). There is regional variation in the age of peak GM volumes, ranging from age 2 to age 10 years (Bethlehem et al. 2022). Therefore, if better EC is associated with either faster or slower development in certain brain regions, the direction of those associations changes in preschool or early school age, complicating the interpretation of results from studies with wide age ranges.

The aim of this study was to address the gaps in the previous literature by utilizing a large cross‐sectional sample of typically developing 5‐year‐old children to examine the neural bases of EC, at an age when both brain and EC are still dynamically developing. This study will provide novel information on the structural associations of EC at this developmentally crucial age. We did not set an explicit hypothesis for this study because there is no previous structural MRI research on EC in this specific age group.

## 2 Methods

This study complies with the Declaration of Helsinki and was approved by the Joint Ethics Committee of the University of Turku and the Hospital District of Southwest Finland (ETMK: 26/1801/2015 for the neuropsychological measurements, and ETMK 31/180/2011 for the neuroimaging).

### 2.1 Participants

Participants were part of the population‐based FinnBrain Birth Cohort Study (www.finnbrain.fi), which is a prospective pregnancy cohort aiming to explore prenatal and early life stress and their effect on child brain development and health (Karlsson et al. 2018). The families were recruited during the mothers' first trimester ultrasound at gestational week (GW) 12, between December 2011 and April 2015 in the South‐Western Hospital District and Åland Islands in Finland. In addition to an ultrasound‐verified pregnancy, sufficient knowledge of the Finnish or Swedish language was required for participation. A written informed consent was provided by the parents prior to the children's study visits.

**FIGURE 1:**
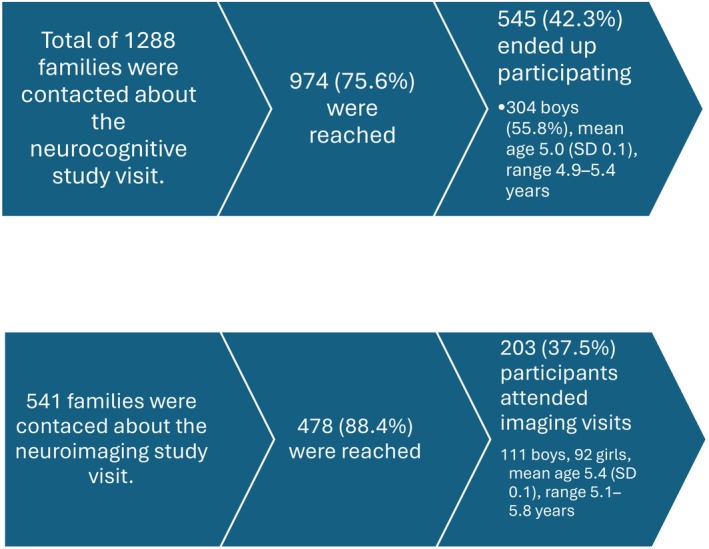
The recruitment process of participants. Participants were healthy, typically developing children, and 182 were right‐handed, 14 left‐handed and 7 had no preference. Mothers of the children who did not participate in the neurocognitive visit (out of the 1288 contacted families) had lower education level (*χ* ^2^ (2) = 30.94, *p* < 0.001), a lower monthly income (*χ* ^2^ (3) = 11.65, *p* = 0.009) and were younger (*t* (1286) = −4.130, *p* < 0.001) compared with the mothers in the families that participated in the neurocognitive visits. Mothers of the children who participated in the neuropsychological visits but not in the neuroimaging visits were older (*t* (369) = 1.97, *p* = 0.047) but did not differ in education level or monthly income compared with the mothers in the families that participated in the MRI visit.

The neurocognitive assessments were conducted at the age of 5 years between October 2017 and December 2020. A total of 1288 families were contacted, from which 545 (42.3%) ended up participating in the neurocognitive study visit (See Figure 1). Next, the families that had attended the neurocognitive visit were prioritized in recruitment for the neuroimaging visits. A total of 196 participants attended both neurocognitive and neuroimaging study visits.

The original goal was to conduct all the neuroimaging study visits when the subjects were between the ages 5 years 3 months and 5 years 5 months; however, some of the participants ended up being older than planned (see Table 1), due to a break in our study visits caused by the COVID‐19 pandemic (152/203 [74.9%] of the participants attended the visit within the intended age range).

**TABLE 1:** Participant demographics and maternal medical history variables (*N* = 155).

Continuous variables	Mean	SD	Min	Max
Age at scan (years)	5.41	0.13	5.08	5.79
Ponderal index^a^	14.05	1.20	11.21	17.63
Gestational age at birth (weeks)	39.80	1.49	33.86	42.29
Birth weight (grams)	3576	467.7	2530	4980
5‐min Apgar score	9.10	0.72	4.00	10.00
Maternal age at term (years)	31.01	4.73	19.10	41.95
Maternal BMI before pregnancy	24.26	4.29	18.13	41.95
Effortful control	5.32	0.58	3.74	6.75
EPDS/SCL‐90 prenatal^b^	22.15	18.68	0	96
EPDS/SCL‐90 postnatal^c^	12.74	13.24	0	70
Delay of Gratification task	5.74	3.12	0	9

Categorical variables	Number	Percent
Sex		
Male	83	53.55
Female	72	46.45
Maternal education level^d^		
Low	31	20.00
Middle	45	29.03
High	79	50.97
Maternal monthly income, estimated after taxes (euros)^e^		
≤ 1500	50	32.26
1501–2500	82	52.90
2501–3500	14	9.03
≥ 3501	2	1.29
Missing	7	4.52
Maternal ethnic background		
Finnish	145	93.55
Other	4	2.58
Missing	6	3.87
GA at birth		
GW ≥ 37	149	96.13
GW < 37	6	3.87
Stay in neonatal intensive care unit		
Yes	23	14.84
No	132	85.16
Alcohol use during pregnancy		
Yes, continued to some degree after learning about pregnancy	21	13.55
Yes, stopped after learning about the pregnancy	21	13.55
No	112	72.26
Missing	1	0.65
Tobacco smoking during pregnancy		
Yes	11	7.10
No	144	92.90
Illicit drug use during pregnancy		
No	146	94.19
Missing	9	5.81
Pregnancy complication^f^		
Yes	24	15.48
No	130	83.87
Missing	1	0.65

Abbreviations: BMI = body mass index; SD = standard deviation.

^a^ Height and weight information was gathered at the neuroimaging visit and used to calculate the ponderal index (weight in kilograms divided by height in meters cubed).

^b^ EPDS/SCL prenatal: Edinburgh Postnatal Depression Scale (EPDS) and Symptom Check List (SCL‐90) scores at GW 14, 24 and 34 were calculated and combined.

^c^ EPDS/SCL postnatal: Combined scores of EPDS and SCL‐90 values at 3‐ and 6‐month check‐ups.

^d^ Maternal education was divided into three classes: Low = upper secondary school or vocational school or lower, Middle = university of applied sciences, High = university; low and middle level education grouped together for statistical analyses.

^e^ The data for maternal monthly income estimate, alcohol use and drug use were acquired at GW 14. Information on maternal age, maternal BMI before pregnancy, birth weight and infant biological sex was obtained from the national birth registries (National Institute for Health and Welfare, www.thl.fi). The tobacco usage variable was created by combining data from our questionnaires and THL: marked as a ‘yes’ if either questionnaire or THL data had a positive for smoking.

^f^ The pregnancy complications variable includes the following ICD‐10 diagnoses: O12 (gestational oedema and proteinuria without hypertension), O13 (gestational hypertension without significant proteinuria), O14 (severe pre‐eclampsia), O24 (diabetes mellitus in pregnancy, childbirth and the puerperium), O46 (antepartum haemorrhage, not elsewhere classified) and O99.0 (anaemia complicating pregnancy, childbirth and the puerperium).

The exclusion criteria for the neuroimaging study were (1) born before GW 35 (before GW 32 for those with exposure to maternal prenatal synthetic glucocorticoid treatment [*n* = 2]); (2) developmental anomaly or abnormalities in senses or communication (e.g., blindness, deafness and congenital heart disease); (3) known long‐term medical diagnosis (e.g., epilepsy and autism); (4) ongoing medical examinations or clinical follow up in a hospital (meaning there has been a referral from primary care setting to special health care); (5) child use of continuous, daily medication (including per oral medications, topical creams and inhalants, apart from desmopressin [Minirin] medication, which was allowed since it is common in the age group and the use of it does not indicate a chronic illness); (6) history of head trauma (defined as concussion necessitating clinical follow up in a health care setting or worse); and (7) metallic (golden) ear tubes (to assure good‐quality scans) and routine MRI contraindications.

Out of the total 196 MR images, 173 were of adequate quality. Of these subjects, 18 parent reports of EC were missing, resulting in a final sample size of 155. The demographic information of the participants is presented in Table 1.

### 2.2 Procedures

The parents reported about child temperament by responding to the Children's Behaviour Questionnaire (CBQ) at 5 years of age during the FinnBrain Child Development and Parenting Functioning Lab's neurocognitive study visit. After that visit, the participating families were invited to the MRI visit, where structural T1‐weighted images were collected as part of a max. 60‐min scan. The study report follows the Strengthening the Reporting of Observational Studies in Epidemiology (STROBE) guidelines (Vandenbroucke et al. 2007). See Supporting Information S2 for the attached checklist.

### 2.3 Neurocognitive Study Visits

The neurocognitive study visits for 5‐year‐old children included neurocognitive testing, eye‐movement tracking, mother–child interaction assessment and questionnaires filled out by the parents, including CBQ. Child EC was assessed with parental reports at the age of 5 via CBQ short form, a revised 94‐item version of the more extensive 195‐item measure (Rothbart et al. 2001). CBQ is a caregiver report measure designed to provide a detailed assessment of temperament in children 3–7 years of age (Rothbart et al. 2001). The parents responded to the CBQ by evaluating their child's behaviour during the past 6 months on a Likert scale from 1–7 (1 being ‘*not at all*’ and 7 being ‘*yes, most of the time*’), with higher scores indicating a higher level of certain characteristics. The CBQ includes 15 dimensions of temperament forming the following main dimensions: EC, negative affectivity and surgency. Our main response variable in this study, EC, consists of four subscales: (1) attentional focusing (ATTFO), the tendency to maintain attentional focus when performing a task; (2) INH, the ability to plan and suppress certain approach responses under instructions; (3) low intensity pleasure (LIP), the pleasure experienced in a situation involving low‐intensity stimuli; and (4) perceptual sensitivity (PER), the detection of low‐intensity stimuli. The statements considering the four subscales of EC were combined, coming to a total of 25 items considering EC and the average was calculated.

EC main dimensions showed good internal consistency, with Cronbach's alpha of 0.829. Cronbach's alpha for the subscales ranged from 0.644 to 0.787, which was considered adequate for the exploratory analyses. One missing answer was allowed (per factor), and the missing one was imputed with the mean of the other answers in the EC questions.

We did not gather data on who filled out the CBQ form; however, the Child Behaviour Checklist (CBCL) form was filled out during the same neurocognitive study visit and included information about the filling parent. Assuming almost complete overlap between CBQ and CBCL fillers, most of the CBCL fillers were mothers (92.90%). In some cases, the filling parent was the father (3.9%), or the parents filled out the form together (1.30%), with data missing from 1.90% of the cases.

The neurocognitive testing included the Delay of Gratification task, which is a delay procedure that measures INH (Beck et al. 2011). The task was presented to the child as a game where they were given a choice between a smaller immediate reward (stickers, treats or coins) and a delayed, larger amount of the same reward type. Choosing the delayed reward was scored as 1 point and the immediate reward as 0 points. The game consisted of nine trials, the scores ranging from 0 to 9, with a higher score indicating a better performance.

### 2.4 Neuroimaging Study Visits

The study visit protocol is described in more depth in previous work from FinnBrain (Copeland et al. 2021; Pulli et al. 2022). Practice materials were delivered to each family prior to the study visit. At the beginning of the study visit, a written informed consent from both parents and a verbal agreement from the child were attained. In the course of the 2‐h preparation time before the scan, the child got familiar with the staff, went through a practice scan and enjoyed a light meal. During the scan, the participants were awake or in natural sleep. Apart from the fMRI sequence, the participants could watch a movie or a cartoon of their choice if they wanted to. A parent and a research staff member were always present in the scanning room throughout the scan. Everyone in the room had their hearing protected with both earplugs and headphones.

### 2.5 MRI Data Acquisition

Siemens Magnetom Skyra fit 3T with a 20‐element head/neck matrix coil was used in scans. The Generalized Autocalibrating Partially Parallel Acquisition (GRAPPA) technique was used to accelerate image acquisition (parallel acquisition technique [PAT] factor of 2 was used). The max. 60‐min scan protocol included a high‐resolution T1 magnetization prepared rapid gradient echo (MPRAGE), a T2 turbo spin echo (TSE), a 7‐min resting state functional MRI and a 96‐direction single shell (*b* = 1000 s/mm^2^) diffusion tensor imaging (DTI) sequence (Kumpulainen et al. 2022; Merisaari et al. 2019; Rosberg et al. 2022) as well as a 31‐direction with *b* = 650 s/mm^2^ and an 80‐direction with *b* = 2000 s/mm^2^. For the purposes of the current study, we acquired high‐resolution T1‐weighted images with the following sequence parameters: TR = 1900 ms, TE = 3.26 ms, TI = 900 ms, flip angle = 9°, voxel size = 1.0 × 1.0 × 1.0 mm^3^, FOV 256 × 256 mm^2^. The scans were planned as per recommendations of the FreeSurfer developers (https://surfer.nmr.mgh.harvard.edu/fswiki/FreeSurferWiki?action=AttachFile&do=get&target=FreeSurfer_Suggested_Morphometry_Protocols.pdf, at the time of writing).

### 2.6 Image Processing

Cortical reconstruction and volumetric segmentation were performed with the Freesurfer 6.0.0 image analysis suite, available for download online (http://surfer.nmr.mgh.harvard.edu/). The technical details of these procedures are described in prior publications (Dale et al. 1999; Dale and Sereno 1993; Fischl et al. 2000, 2001, 2002; Fischl, Salat, et al. 2004; Fischl, Sereno, and Dale 1999; Fischl, Sereno, Tootell, and Dale 1999; Fischl, van der Kouwe, et al. 2004; Han et al. 2006; Jovicich et al. 2006; Reuter et al. 2010, 2012; Ségonne et al. 2004).

After the initial FreeSurfer processing, all images were visually checked and manually edited (skull fragments removed if they were influencing the pial border, errors corrected in the border between GM and white matter and arteries removed). After the edits, the FreeSurfer recon‐all was run once more. For a more detailed description of the image processing procedure, please see our previous article (Pulli et al. 2022).

### 2.7 Covariate Selection for Vertex‐Wise Statistics

The potential confounders were chosen based on prior evidence on their associations with cortical brain structure in 5‐year‐olds (Silver et al. 2022). Covariates in our analyses were the child's sex, age at scan, ponderal index (mass in kilograms divided by height in meters cubed; measured during the neuroimaging visit), maternal age at term and maternal education level. These variables are significant predictors for cortical surface area and volume, reflecting both biological and environmental influences (Silver et al. 2022). Maternal education data were gathered from questionnaire data from GW 14 or 5 years of child age by using the highest degree reported (two classes, university vs. other degree).

### 2.8 Sensitivity Analyses

We performed sensitivity analyses controlling for the following factors: maternal body mass index (BMI) before pregnancy (Li et al. 2016; Ou et al. 2015) and GWs at birth. In other sensitivity analyses, a part of the sample was excluded: We took into account alcohol exposure in utero (Donald et al. 2015), tobacco exposure (Chang et al. 2016; Knickmeyer et al. 2017) and children with neonatal intensive care unit (NICU) stay were excluded (Aoki et al. 2020). We also controlled for maternal scores from the Edinburgh Postnatal Depression Scale (EPDS) (Cox et al. 1987) and Symptom Checklist 90 (SCL‐90) (Derogatis 1994). The detailed models are presented in the Supporting Information.

### 2.9 Data Sharing Statement

Current EU and national legislation on personal data protection of sensitive data and the informed consents given by the study subjects do not permit open data sharing. Investigators interested in research collaboration and obtaining access to the data are encouraged to contact the FinnBrain board (finnbrain-board@lists.utu.fi). Contact information of the board members or FinnBrain's Principal Investigators are listed on the project website: https://sites.utu.fi/finnbrain/en/contact/.

## 3 Statistics

IBM SPSS for Mac, version 27.0 (IBM Corp., Armonk, NY, USA) was used for the statistical analyses concerning demographics.

As instructed in the FreeSurfer manual, we pre‐smoothed fsaverage surfaces for analyses with Query, Design, Estimate, Contrast (Qdec), a single‐binary application included in the FreeSurfer software suite (http://surfer.nmr.mgh.harvard.edu/). Qdec is a graphical user interface for a statistics engine running a vertex‐by‐vertex general linear model (GLM). For display purposes, we used the standard FreeSurfer's fsaverage in MNI305 space (MNI = Montreal Neurological Institute).

We tested for clusters with statistically significant associations between EC and cortical GM volume, surface area and cortical thickness. In addition to EC, we performed post hoc analysis on all its subscales (ATTFO, INH, LIP and PER).

The data were smoothed with a kernel of 10 mm full width at half maximum. A Monte Carlo null‐*Z* simulation was run with a *z*‐value threshold of 1.3, corresponding to *p* = 0.05 (Hagler et al. 2006). Monte Carlo null‐*Z* simulation is commonly used in voxel‐wise analyses for cluster‐level inference. For the performed sensitivity analyses, please see Section 2.7. Age at scan was squared for the purposes of running Qdec.

## 4 Results

### 4.1 EC and Brain Volume

**FIGURE 2:**
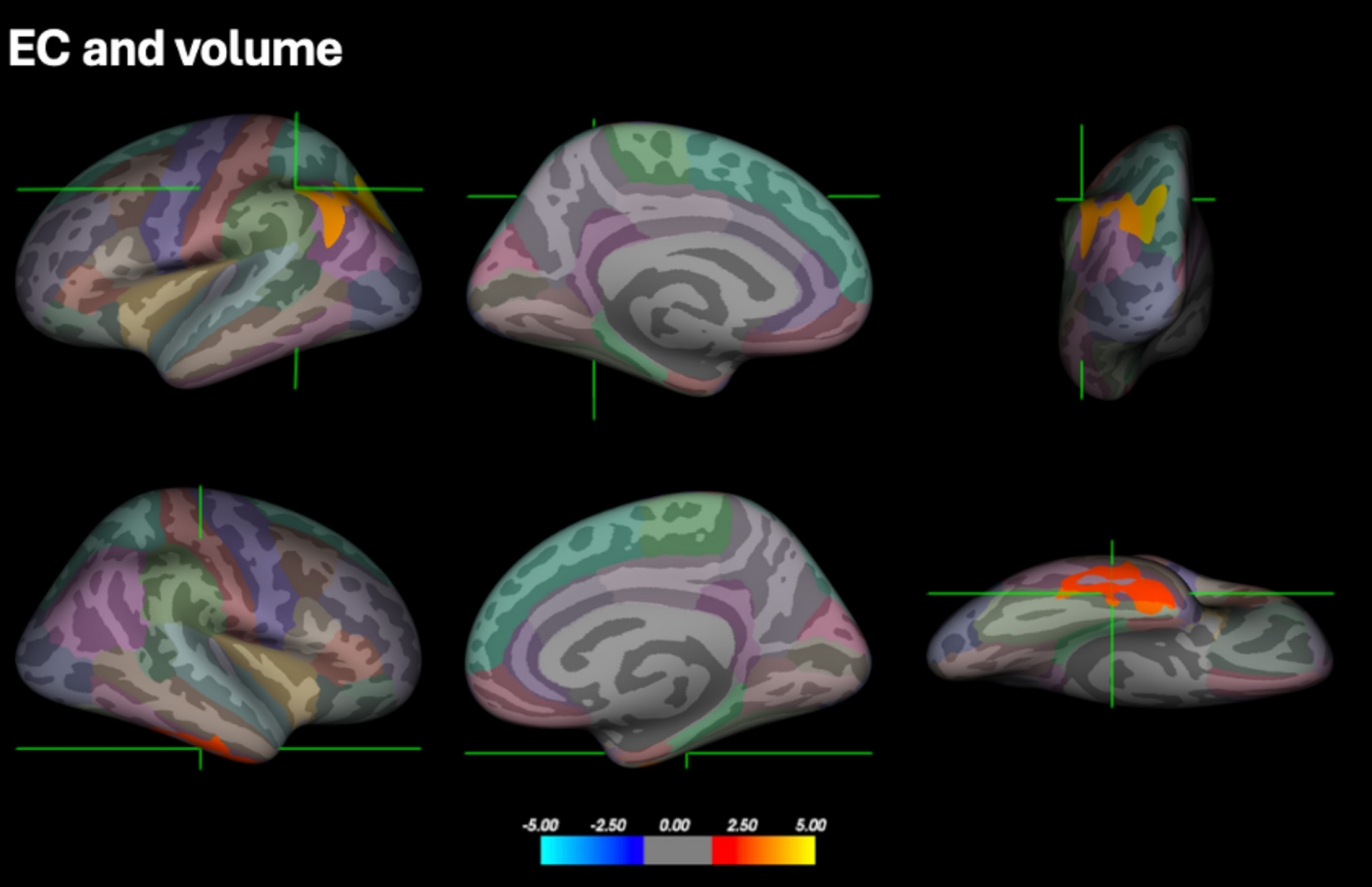
Positive associations between EC and cortical volume. Result corrected for multiple comparisons using Monte Carlo null‐*Z* simulation. The position of the green crosshair indicates the most statistically significant vertex in statistically significant clusters. The left hemisphere is pictured on the top row, and the right hemisphere on the bottom row. From left to right, first is lateral and second medial view. The last pictures on the right, where both clusters are best visible, are posterior view of the left hemisphere and basal view of the right hemisphere. Colour coding of regions according to the Desikan–Killiany atlas. Scale: −log_10_(*p*).

Figure 2 presents the associations between brain volume and EC. The significant associations were positive. On the left hemisphere, there was a cluster in the supramarginal region (peak *p* < 0.0001, size: 1961.59 mm^2^, peak coordinates: −49.6, −48.0, 44.6). On the right hemisphere, there was a cluster in the inferior temporal region (peak *p* = 0.0025, size: 1253.93 mm^2^, peak coordinates: 46.9, −21.1, −26.6).

### 4.2 EC and Surface Area

**FIGURE 3:**
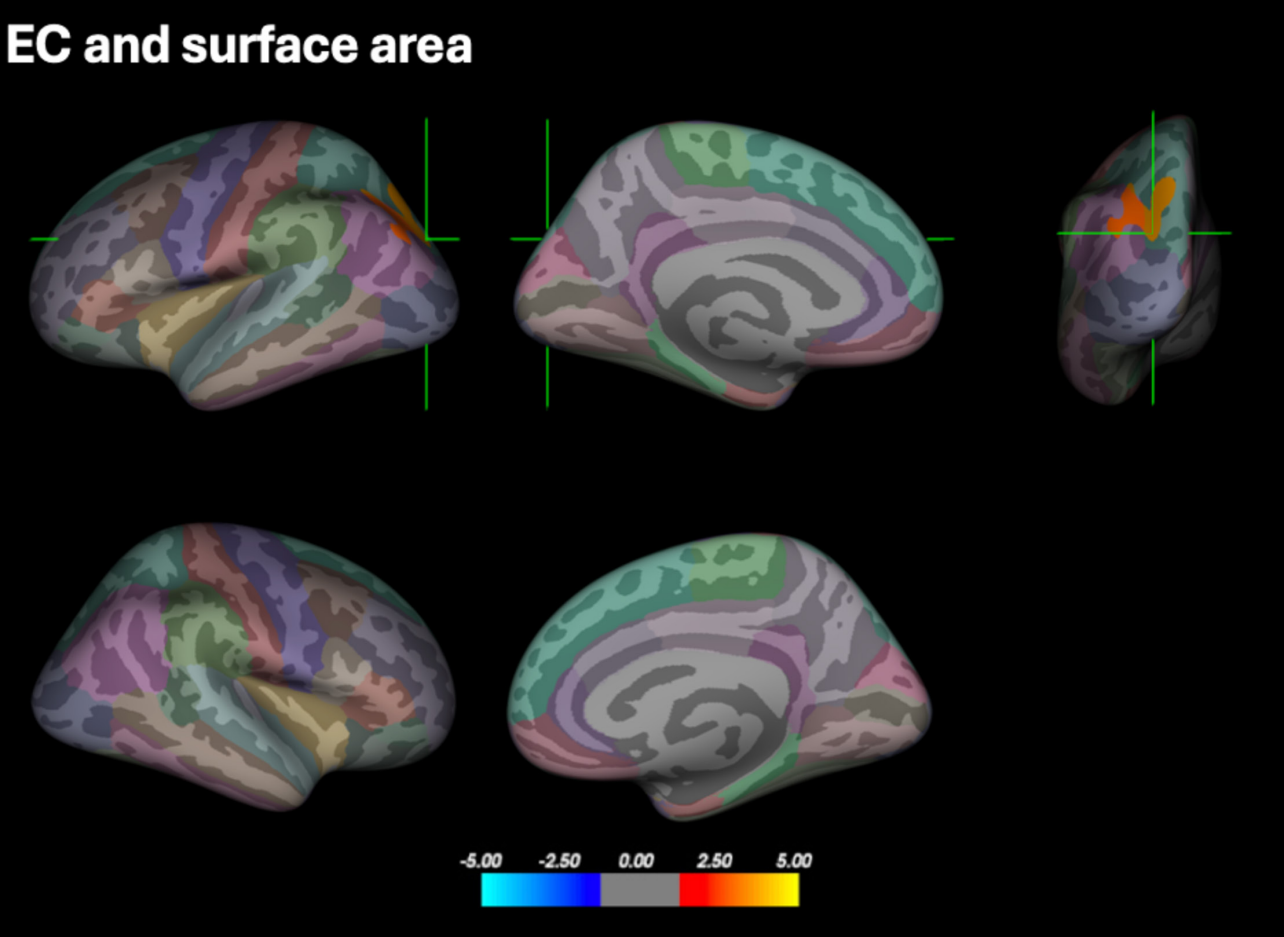
Positive association between EC and surface area. Result corrected for multiple comparisons using Monte Carlo null‐*Z* simulation. The position of the green crosshair indicates the most statistically significant vertex in statistically significant clusters. The left hemisphere is pictured on the top row, and the right hemisphere on the bottom row. The first picture from left to right is lateral view and the second is medial view. On the top right corner, a posterior view of the left hemisphere is included. Colour coding of regions according to the Desikan–Killiany atlas. Scale: −log_10_(*p*).

The cluster coordinates of the positive association in the left hemisphere were the same between EC and surface area, INH and surface area, and LIP and volume in the superior parietal region. Figure 3 presents the associations between surface area and EC, where the only significant association was positive. The cluster was located on the left hemisphere in the superior parietal region (peak *p* = 0.0007, size: 1202.95 mm^2^, peak coordinates: −23.9, −78.5, 16.8). No clusters were found on the right hemisphere.

### 4.3 EC and Cortical Thickness

We also analysed associations between EC and cortical thickness on Qdec, using the same variables, but no clusters were found after conducting the Monte Carlo simulation.

### 4.4 Post Hoc Analyses

**FIGURE 4:**
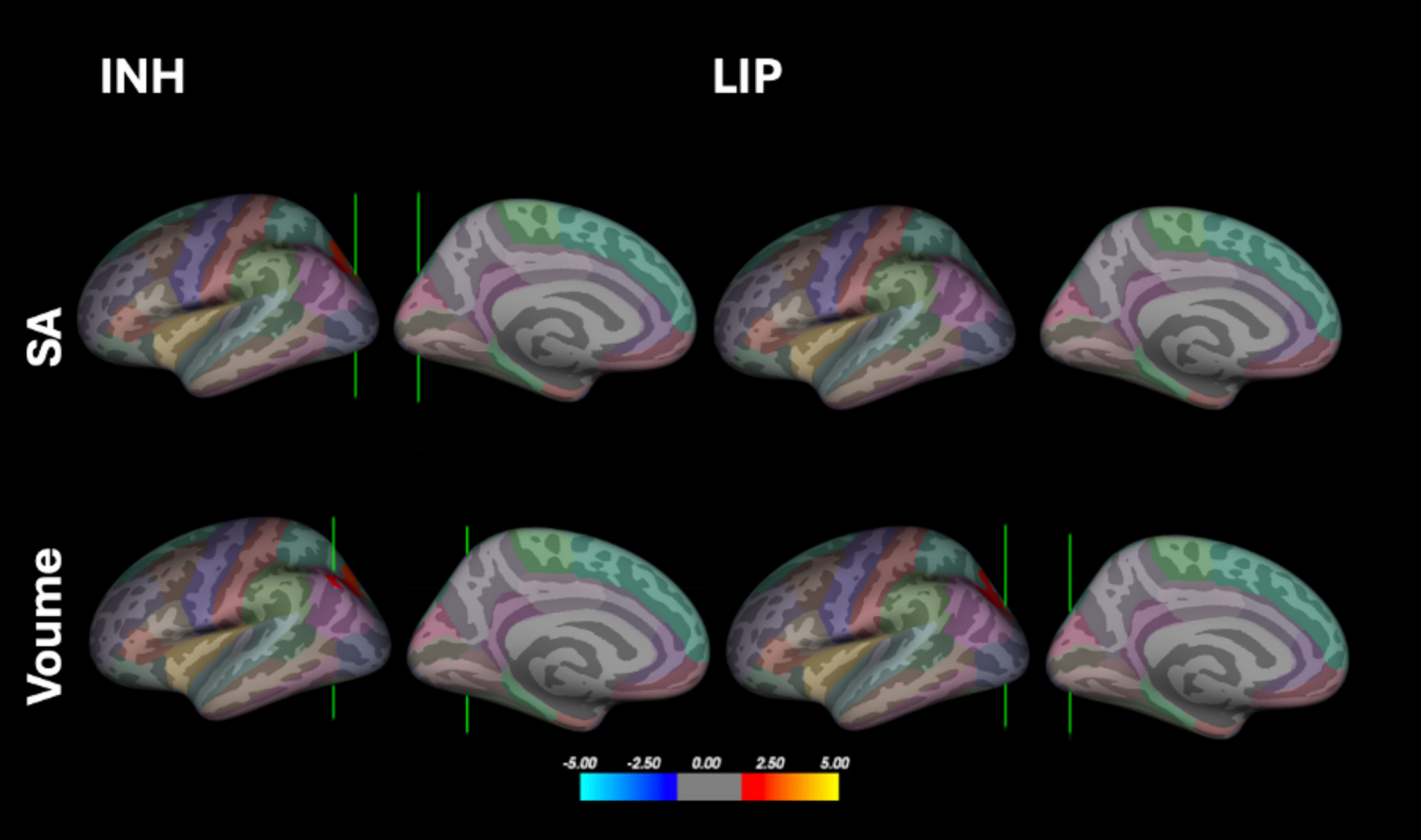
The association between inhibitory control (INH) and low intensity pleasure (LIP) with surface area on the top row and with volume on the bottom row. Both lateral and medial views of the left hemisphere are included. The right hemisphere is not included, since no significant associations were found. Result corrected for multiple comparisons using Monte Carlo null‐*Z* simulation. The position of the green crosshair indicates the most statistically significant vertex in statistically significant clusters. Colour coding of regions according to the Desikan–Killiany atlas. Scale: −log_10_(*p*).

We further explored post hoc analyses to determine whether specific subscales of EC accounted for the observed results. Associations were only found between neural correlates and INH and LIP; all significant associations were positive and only visible on the left hemisphere (Figure 4).

Cluster representing the association between INH and surface area was in the superior parietal region (peak *p* = 0.0200, size: 786.69 mm^2^, peak coordinates: −23.9, −78.5, 16.8), whereas the cluster representing the association between INH and brain volume was in the inferior parietal region (peak *p* = 0.0151, size: 989.46 mm^2^, peak coordinates: −37.8, −67.0, 45.6).

No cluster was found when analysing the association between LIP and surface area. The cluster representing the association between LIP and volume was in the superior parietal region (peak *p* = 0.0417, size: 851.96 mm^2^, peak coordinates: −23.9, −78.5, 16.8).

We also analysed the association between the Delay of Gratification task (Beck et al. 2011) and the 5‐year‐olds' MRI data. However, there were no results found when removing the unreliable data. The analysis with the entire sample and an explanation of the exclusions were included in the Supporting Information (see Figure S4).

### 4.5 Sensitivity Analyses

No major differences were found in the sensitivity analyses. The results are presented in detail in the Supporting Information.

## 5 Discussion

In this study, we investigated the associations between EC and structural cortical brain metrics, more precisely volume, surface area and cortical thickness, in a large sample of typically developing 5‐year‐olds. We also examined the subscales of EC and their relation to cortical brain anatomy, to assess whether the effects were driven by any specific subscales. All significant findings were positive, and almost all significant results were found on the left hemisphere. Higher EC (and its subscales) was generally related to higher volume or larger surface area in the child's brain.

No significant associations between EC and cortical thickness were found. A possible reason for that is that the differences in cortical thickness are quite small. Therefore, the volume is mainly determined by surface area, therefore explaining associations observed in these two metrics. Because of the smaller variability, cortical thickness in comparison with surface area may also be more sensitive to motion artefact, which has been linked to the loss of GM volume (Blumenthal et al. 2002).

To summarize the results, higher child EC was associated with a cluster located in the left superior parietal, left supramarginal and right inferior temporal region. Based on post hoc analysis, these associations were driven by EC indicators of LIP and INH specifically. The parietal regions in our results are integral parts of the network responsible for top‐down regulation: the fronto‐parietal network. The fronto‐parietal network has been linked to executive functions, including INH; see review by Fiske and Holmboe (2019). This network may partly account for the previously mentioned association between EC and academic success (Robson et al. 2020). The superior parietal gyrus is involved in attention orientation (Salmi et al. 2007), which plays an important role in SR, particularly at this young age. Our findings may reflect the developmental transition in the neural basis of SR: Early SR relies more on the orienting networks, whereas the executive networks start to play a more integral part from 4 years of age onwards, with their contribution continuing to increase across development (Posner et al. 2014). Accordingly, the neuroanatomical basis of SR is likely to differ in older children, where executive networks dominate over the orienting networks, partially explaining the differences between our results and previous literature.

Though direct associations between EC and the parietal lobe have not been widely reported previously, there are reports of higher parietal lobe volume linking with conscientiousness (Wang et al. 2019), which is considered a continuum for higher childhood SR (and thus, EC). In addition, parietal regions have previously been linked to task‐switching (Wong and Yu 2024) and orienting attention (Wager et al. 2004), both of which are central components in SR. Furthermore, the parietal lobe is part of the parieto–frontal integration theory (P‐FIT); see review by Jung and Haier (2007), which describes a network supporting higher order cognitive processes, similar to executive function, a concept closely related to SR and INH; see review by Fiske and Holmboe (2019).

Child brain development is rapid and region‐specific. At 5 years of age, the GM volume is close to reaching its peak, which happens at 5.9 years (Bethlehem et al. 2022; Courchesne et al. 2000). However, the regional peaks of GM volume in the cortical regions vary notably between ages 2 and 10 years (Bethlehem et al. 2022) and occipito–parietal volumes peak first before the age of 5 years (Aubert‐Broche et al. 2013; Bethlehem et al. 2022). The correlations found by previous research on older participants are located more anteriorly, in an area that reaches its GM volume peak later. This could suggest that the region linked to SR changes through development. The cluster found includes regions that peak in volume around or before 5 years of age, making it difficult to conclude whether the children with higher EC are further along in brain development or developing more slowly. We found a positive correlation between EC and right inferior temporal surface area, a region still growing towards its peak (Bethlehem et al. 2022), which suggests that EC is higher in children who are further along in brain development. Whereas the positive result in the parietal region is not as straightforward to interpret.

Longitudinal studies with repeated imaging that allow comparing the same individuals at different age points close to each other will be crucial in identifying the neural underpinnings of EC at different stages of development. In addition to within‐individual tracking of brain maturation and EC assessments, meta‐analytic approaches combining studies with narrow age ranges may offer additional insight into the developmental trajectories.

In addition to questionnaires, we examined the neural correlates of the ability to delay gratification. The results were observed with questionnaire‐based data, but not with task‐based evaluation of SR. Therefore, the findings may specifically reflect the everyday context of SR rather than the more narrowly defined part of regulatory processes observed in a laboratory setting (Hardikar et al. 2024). This difference in results exemplifies the importance of using complementary questionnaires and task‐based measurements to get a more complete image of the individual's SR and the underlying neural basis.

We found no supporting evidence for the links between EC and either PFC or ACC, which are the most important neural correlates indicated in the previous studies (Kelley et al. 2015) and parts of the P‐FIT (Jung and Haier 2007). In our study, none of the results in the OFC survived the Monte Carlo simulation, and no negative results survived altogether. Previous MRI studies have found a positive correlation between left OFC volume and EC in 12‐year‐olds (Whittle et al. 2008). This same correlation was also found in a lesion study on adults by Bechara et al. (2000), and a similar result was found by Hadaya et al. (2023), where in 4–7‐year‐olds, higher bilateral OFC and left insula volume and centrality in the left OFC were linked to higher EC. Furthermore, like in previous studies, most findings in our study were found in the left hemisphere, but the specific regions differed significantly.

One possible reason for the discrepancies between the previous research and our study may lie in the age differences between the studies. From the perspective of rapid and region‐specific child brain development, the age difference of 7 years (compared with the study population in Whittle et al. 2008) is substantial, especially given how puberty significantly shapes brain trajectories. Brain–behaviour relationships can change with age, depending on the measurement and developmental stage, and either faster or slower neurodevelopment can be beneficial (Callaghan and Tottenham 2016; Shaw et al. 2006; Squeglia et al. 2013).

As stated above, the regions identified in our results include areas that peak around or before our participants' mean age. This makes it difficult to interpret whether larger neuroanatomical structures at this stage reflect accelerated or delayed brain maturation. These findings illustrate brain–behaviour associations at this age point, and the results may not be comparable with those in older children. Similar structural patterns can reflect different developmental processes at different age points.

Other factors that might explain variation in results are that different studies use different versions of Rothbart's questionnaire battery, younger children typically being rated by parents, whereas older children may self‐report EC. For example, Cheetham et al. (2017) and Whittle et al. (2008) both used the self‐reported Early Adolescent Temperament Questionnaire Revised, which is a temperament and SR assessment tool for adolescents (Capaldi and Rothbart 1992).

This study has some limitations. First, the narrow age range is mostly a strength of this study, but the absence of longitudinal data is a limitation. A cross‐sectional study with a relatively large sample provides reliable information at this specific age point, whereas a wider age range could cover different stages of development. Different questionnaire versions may also be based on slightly different items of factor structure and may link differently with neural features. Furthermore, some of the earlier studies used task‐based assessment of EC (e.g., INH tasks) that may tap into different aspects of EC compared with the parental report. Second, even though the EC trait is universal and shared across cultures, the developmental outcomes (especially the ones rated using questionnaires) may vary between cultures, depending on which behaviours are valued in the society (Rothbart 2007). This study focuses solely on Finnish 5‐year‐olds (many of whom are from a high socio‐economic background), and the results may not generalize to other cultures and countries. Finally, the reliance on parental report is suboptimal because of its subjectivity. Parents can have biased views of their children, and the parents' own traits and abilities affect the replies. Parental reports may also bias the answers towards outwardly visible behaviours, whereas self‐reports may be able to capture more nuanced inter‐individual differences.

## 6 Conclusions

Early EC and self‐regulatory traits are important predictors for many social and health outcomes later in life. Characterizing the early neural correlates of EC provides a necessary foundation for future longitudinal work examining developmental trajectories of SR and related outcomes. In this study, we extend the prior literature by examining links between several structural brain features simultaneously (volumes, surface area and cortical thickness) and child EC in typically developing 5‐year‐olds.

The novelty in this study is in its large sample size within a narrowly defined age range. This reduces developmental variability between the participants and enables a more precise assessment of brain–behaviour associations in this sample of typically developing 5‐year‐olds. Furthermore, both questionnaire and task‐based measurements were applied, and only questionnaire data were associated with brain structure. The regions that were significant in this study, especially the left parietal lobe, have not been linked to EC before. There are at least two possible explanations. First, there are the methodological differences of sample age and the EC assessment tool used in some of the previous studies investigating the structural cortical development of small children in the context of EC development. Second, this could be an age‐specific finding. It is possible that the ability to self‐regulate and to make plans is reflected in the parietal lobe at this stage of development, but with age, other regions become increasingly important. Longitudinal studies will be crucial in exploring this possibility and the developmental outcomes further.

## Author Contributions

**Meri Frantti:** conceptualization, formal analysis, visualization, writing – original draft. **Jetro J. Tuulari:** conceptualization, supervision, methodology, writing – review and editing. **Saara Nolvi:** conceptualization, writing – original draft. **Elisabeth Nordenswan:** investigation, writing – review and editing. **Anni Copeland:** investigation, writing – review and editing. **Venla Kumpulainen:** investigation, writing – review and editing. **Eero Silver:** investigation, writing – review and editing. **Harri Merisaari:** resources, writing – review and editing. **Ekaterina Saukko:** investigation, writing – review and editing. **Eeva‐Leena Kataja:** writing – review and editing. **Riikka Korja:** funding acquisition, writing – review and editing. **Linnea Karlsson:** funding acquisition, project administration, writing – review and editing. **Hasse Karlsson:** funding acquisition, project administration, writing – review and editing. **Elmo P. Pulli:** conceptualization, formal analysis, supervision, methodology, writing – original draft.

## Funding

This work was supported by the Alfred Kordelinin Säätiö, Emil Aaltosen Säätiö, Juho Vainion Säätiö, Research Council of Finland (26080983, 346121, 352648), Sigrid Juséliuksen Säätiö, Varsinais‐Suomen Sairaanhoitopiiri, Turun Yliopistosäätiö, Signe ja Ane Gyllenbergin Säätiö, Orionin Tutkimussäätiö, Finnish State Grants for Clinical Research (ERVA/VTR), The Centre for Exellence, Frilasarettet Eschnerska Stiftelsen, Päivikki and Sakari Sohlberg Foundation, Finnish Brain Foundation, Finnish Cultural Foundation and Academy of Finland (346790).

## Conflicts of Interest

The authors declare no conflicts of interest.

## Supporting information


**Figure S1:** The correlation between effortful control (EC) and cortical volume on the left hemisphere. Covariates in all analyses were the child's gender, age at scan, ponderal index (mass in kilograms divided by height in meters cubed; measured during the neuroimaging visit), maternal age at term and maternal education level. In sensitivity analyses, an additional factor was controlled for: maternal body mass index (BMI) before pregnancy, gestational weeks (GW) at term and both maternal prenatal and postnatal scores of Edinburgh Postnatal Depression Scale (EPDS) and Symptom Checklist 90 (SCL‐90). In other sensitivity analyses, a part of the sample was excluded: alcohol exposure in utero with those excluded, whose mothers continued drinking after learning about pregnancy (*n* = 134), tobacco exposure in utero, the ones with exposure excluded (*n* = 144) and children with neonatal intensive care unit (NICU) stay excluded (*n* = 132). Cluster colour indicates significance as a *z*‐value. The position of the green crosshair indicates the most statistically significant vertex in statistically significant clusters. Colour coding of regions according to the Desikan–Killiany atlas. No correction for multiple comparisons was made.
**Figure S2:** The correlation between EC and cortical volume on the right hemisphere. Covariates in all analyses were the child's gender, age at scan, ponderal index (mass in kilograms divided by height in meters cubed; measured during the neuroimaging visit), maternal age at term and maternal education level. In sensitivity analyses, an additional factor was controlled for maternal body mass index (BMI) before pregnancy, gestational weeks (GW) at term and both maternal prenatal and postnatal scores of Edinburgh Postnatal Depression Scale (EPDS) and Symptom Checklist 90 (SCL‐90). In other sensitivity analyses, a part of the sample was excluded: alcohol exposure in utero with those excluded, whose mothers continued drinking after learning about pregnancy (n = 134), tobacco exposure in utero, the ones with exposure excluded (*n* = 144) and children with neonatal intensive care unit (NICU) stay excluded (n = 132). Cluster colour indicates significance as a z‐value. The position of the green crosshair indicates the most statistically significant vertex in statistically significant clusters. Colour coding of regions according to the Desikan–Killiany atlas. No correction for multiple comparisons was made.
**Figure S3:** The correlation between EC and brain surface area (SA) on the left hemisphere. No significant clusters were found between EC and SA in our main results in the right hemisphere. The basic model (N = 155) in the top left corner has been corrected for child's gender, age at scan, ponderal index (mass in kilograms divided by height in meters cubed; measured during the neuroimaging visit), maternal age at term and maternal education level. In sensitivity analyses, an additional factor was controlled for: maternal body mass index (BMI) before pregnancy, gestational weeks (GW) at term and both maternal prenatal and postnatal scores of Edinburgh Postnatal Depression Scale (EPDS) and Symptom Checklist 90 (SCL‐90). In other sensitivity analyses, a part of the sample was excluded: alcohol exposure in utero with those excluded, whose mothers continued drinking after learning about pregnancy (n = 134), tobacco exposure in utero, the ones with exposure excluded (n = 144) and children with neonatal intensive care unit (NICU) stay excluded (n = 132). Cluster colour indicates significance as a z‐value. The position of the green crosshair indicates the most statistically significant vertex in statistically significant clusters. Colour coding of regions according to the Desikan–Killiany atlas. No correction for multiple comparisons was made.
**Figure S4:** The correlation between the Delay of Gratification task and cortical volume presented on the top row, surface area in the middle and cortical thickness on the bottom row (n = 165). The results were corrected for child's gender, age at scan, ponderal index (mass in kilograms divided by height in meters cubed; measured during the neuroimaging visit), maternal age at term and maternal education level. There were no results found when the unreliable data were removed. There were 11 cases of unreliable data due to the following four reasons: (1) testing related, e.g., researcher error and incorrect task presentation; (2) child related, e.g., restlessness and inability to follow orders; (3) parent present; and (4) other. Colour coding of regions according to the Desikan–Killiany atlas. No correction for multiple comparisons was made.

**Data S1:** We conducted a Pubmed search on 21 January 2024 with the following query: mri AND (structur* OR volume OR area OR ‘cortical thickness’) AND (infan* OR toddler OR child OR childre* OR adolesc*) AND (self‐control OR ‘effortful control’ OR ‘executive function’ OR ‘inhibitory control’) and the last two articles on the table are added from our previous searches.

**Data S2:** Supporting information.
