# Supplementary material for: Effortful Control and Cortical Brain Structure in 5‐Year‐Old Children: Findings From the FinnBrain Birth Cohort Study
Source: Eur J Neurosci. 2026 Jun 10;63(11):e70580. doi: 10.1111/ejn.70580 (PMC13250741; doi:10.1111/ejn.70580)
Supplement: Supplementary file 1 — Figure S1: The correlation between effortful control (EC) and cortical volume on the left hemisphere. Covariates in all analyses were the child's gender, age at scan, ponderal index (mass in kilograms divided by height in meters cubed; measured during the neuroimaging visit), maternal age at term and maternal education level. In sensitivity analyses, an additional factor was controlled for: maternal body mass index (BMI) before pregnancy, gestational weeks (GW) at term and both maternal prenatal and postnatal scores of Edinburgh Postnatal Depression Scale (EPDS) and Symptom Checklist 90 (SCL‐90). In other sensitivity analyses, a part of the sample was excluded: alcohol exposure in utero with those excluded, whose mothers continued drinking after learning about pregnancy (n = 134), tobacco exposure in utero, the ones with exposure excluded (n = 144) and children with neonatal intensive care unit (NICU) stay excluded (n = 132). Cluster colour indicates significance as a z‐value. The position of the green crosshair indicates the most statistically significant vertex in statistically significant clusters. Colour coding of regions according to the Desikan–Killiany atlas. No correction for multiple comparisons was made. Figure S2: The correlation between EC and cortical volume on the right hemisphere. Covariates in all analyses were the child's gender, age at scan, ponderal index (mass in kilograms divided by height in meters cubed; measured during the neuroimaging visit), maternal age at term and maternal education level. In sensitivity analyses, an additional factor was controlled for maternal body mass index (BMI) before pregnancy, gestational weeks (GW) at term and both maternal prenatal and postnatal scores of Edinburgh Postnatal Depression Scale (EPDS) and Symptom Checklist 90 (SCL‐90). In other sensitivity analyses, a part of the sample was excluded: alcohol exposure in utero with those excluded, whose mothers continued drinking after learning about pregnancy (n = [file EJN-63-0-s002.docx]

Effortful control and cortical brain structure in 5-year-old children: findings from FinnBrain Birth Cohort

Authors: Meri Frantti, Jetro J. Tuulari, Saara Nolvi, Elisabeth Nordenswan, Anni Copeland, Venla Kumpulainen, Eero Silver, Harri Merisaari, Ekaterina Saukko, Eeva-Leena Kataja, Riikka Korja, Linnea Karlsson, Hasse Karlsson, Elmo P. Pulli

### Sensitivity analyses

We performed sensitivity analyses controlling for the following factors that are associated with altered brain development either by controlling for them in the analyses (continuous variables) or by excluding those with exposure from analyses (categorical variables). Maternal body mass index (BMI) before pregnancy (Li et al., 2016; Ou et al., 2015) and gestational weeks at birth (Jeong et al., 2016; Kapellou et al., 2006) were added as covariates. In other sensitivity analyses, a part of the sample was excluded: we took into account alcohol exposure in utero (missing data interpreted as no exposure) (Donald et al., 2015), tobacco exposure (Chang et al., 2016; Knickmeyer et al., 2016) and children with neonatal intensive care unit (NICU) stay were excluded (Aoki et al., 2020). We also controlled for maternal scores from the Edinburgh Postnatal Depression Scale (EPDS), a self-report questionnaire of depressive symptoms within the past 7 days (Cox et al., 1987), and Symptom Checklist 90 (SCL-90), an assessment of psychiatric symptoms within the past month (Derogatis, 1994). We calculated prenatal score combining EPDS and SCL-90 scores from GW 14, 24 and 34. Postnatal value consisted of EPDS and SCL-90 values at 3- and 6-monts of child age. The EPDS consists of 10 items considering 3 subscales: anxiety, anhedonia and depression, and the total score ranges from 0 to 30, larger score meaning more depressive symptoms. We used the Anxiety Subscale of the SCL-90, score ranging from 0–40, higher score meaning more severe symptoms. No mothers reported usage of drugs based on self-report.


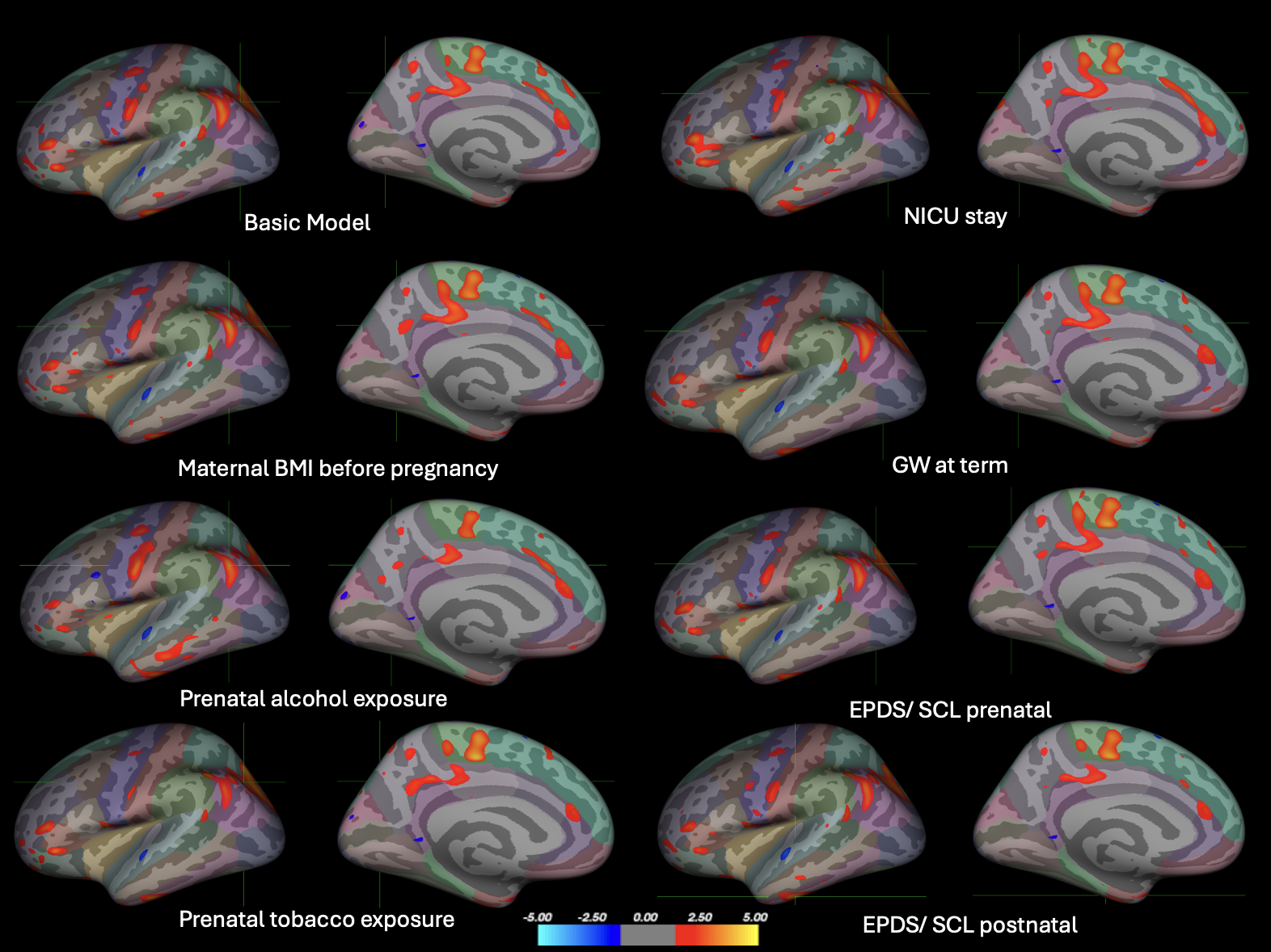


Supplementary Figure 1: The correlation between Effortful Control (EC) and cortical volume on the left hemisphere. Covariates in all analyses were the child’s gender, age at scan, ponderal index (mass in kilograms divided by height in meters cubed; measured during the neuroimaging visit), maternal age at term and maternal education level. In sensitivity analyses an additional factor was controlled for: maternal body mass index (BMI) before pregnancy, gestational weeks (GW) at term and both maternal prenatal and postnatal scores of Edinburgh Postnatal Depression Scale (EPDS) and Symptom Checklist 90 (SCL-90). In other sensitivity analyses, a part if the sample was excluded: alcohol exposure in utero with those excluded, whose mothers continued drinking after learning about pregnancy (n=134), tobacco exposure in utero, the ones with exposure excluded (n=144) and children with neonatal intensive care unit (NICU) stay excluded (n=132). Cluster color indicates significance as a z-value. The position of the green crosshair indicates the most statistically significant vertex in statistically significant clusters. Color coding of regions according to the Desikan-Killiany atlas. No correction for multiple comparisons was made.


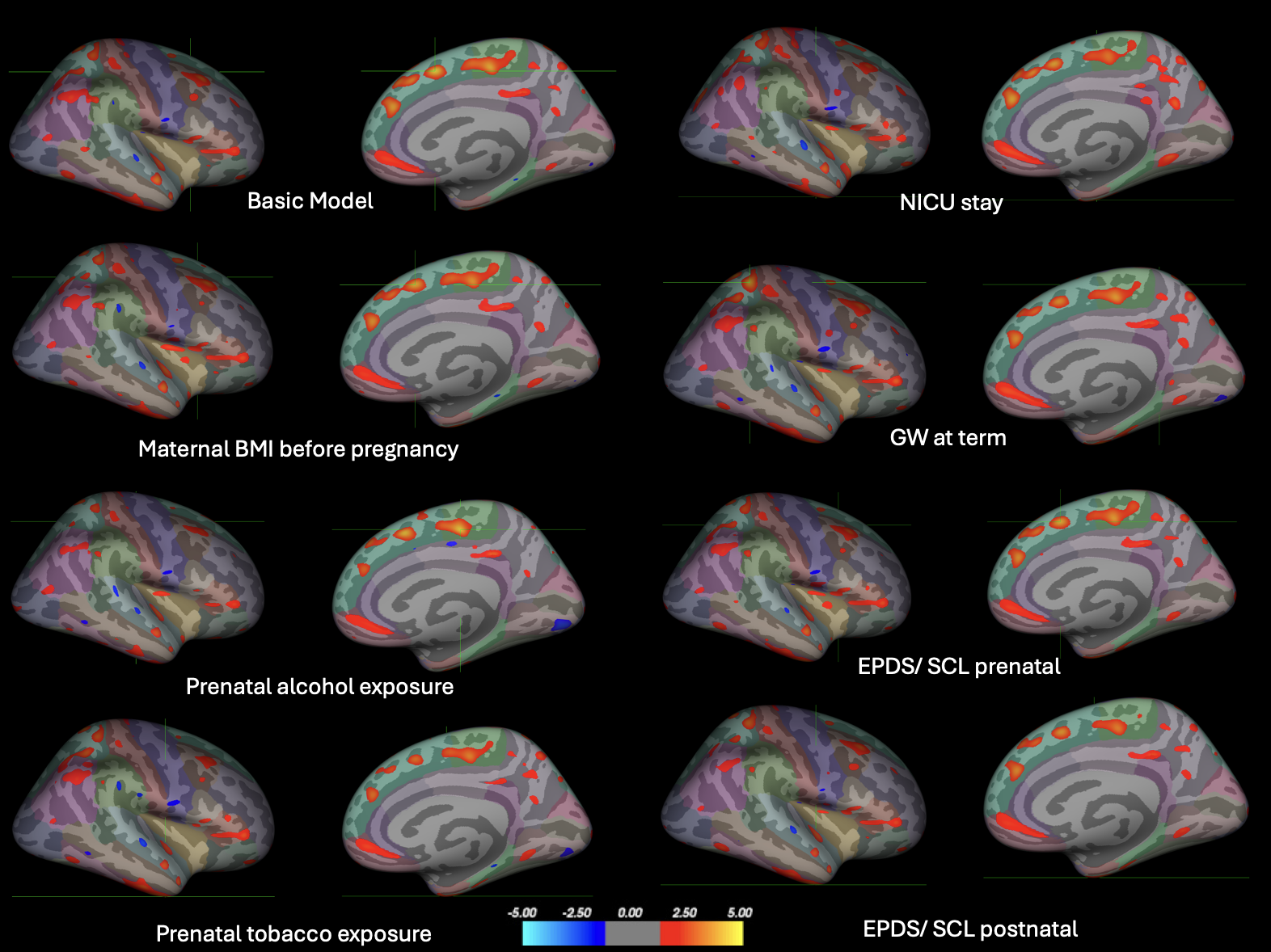


Supplementary Figure 2: The correlation between EC and cortical volume on the right hemisphere. Covariates in all analyses were the child’s gender, age at scan, ponderal index (mass in kilograms divided by height in meters cubed; measured during the neuroimaging visit), maternal age at term and maternal education level. In sensitivity analyses an additional factor was controlled for: maternal body mass index (BMI) before pregnancy, gestational weeks (GW) at term and both maternal prenatal and postnatal scores of Edinburgh Postnatal Depression Scale (EPDS) and Symptom Checklist 90 (SCL-90). In other sensitivity analyses, a part if the sample was excluded: alcohol exposure in utero with those excluded, whose mothers continued drinking after learning about pregnancy (n=134), tobacco exposure in utero, the ones with exposure excluded (n=144) and children with neonatal intensive care unit (NICU) stay excluded (n=132). Cluster color indicates significance as a z-value. The position of the green crosshair indicates the most statistically significant vertex in statistically significant clusters. Color coding of regions according to the Desikan-Killiany atlas. No correction for multiple comparisons was made.


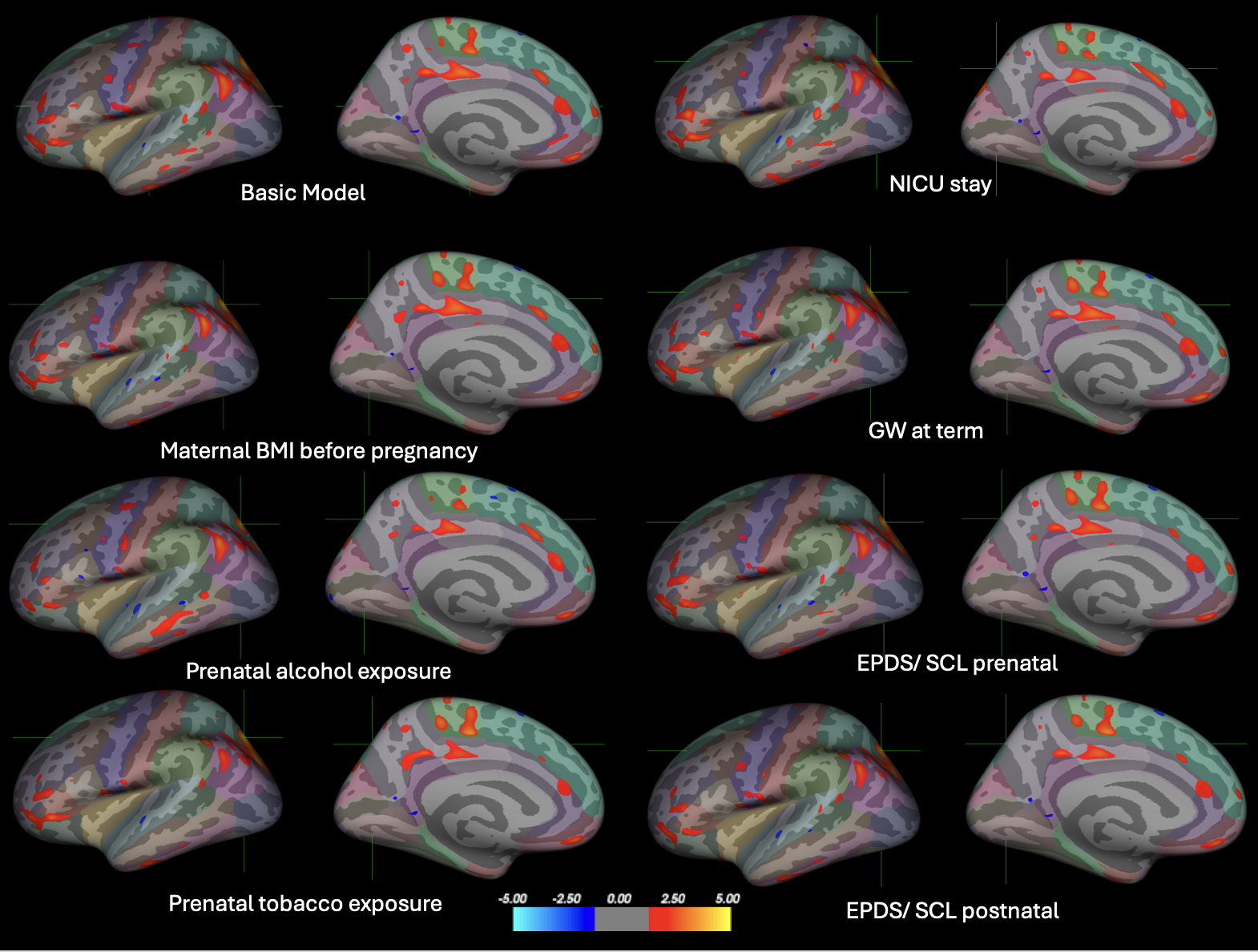


Supplementary Figure 3: The correlation between EC and brain surface area (SA) on the left hemisphere. No significant clusters were found between EC and SA in our main results in the right hemisphere. The basic model (N=155) in the top left corner has been corrected for child’s gender, age at scan, ponderal index (mass in kilograms divided by height in meters cubed; measured during the neuroimaging visit), maternal age at term and maternal education level. In sensitivity analyses an additional factor was controlled for: maternal body mass index (BMI) before pregnancy, gestational weeks (GW) at term and both maternal prenatal and postnatal scores of Edinburgh Postnatal Depression Scale (EPDS) and Symptom Checklist 90 (SCL-90). In other sensitivity analyses, a part if the sample was excluded: alcohol exposure in utero with those excluded, whose mothers continued drinking after learning about pregnancy (n=134), tobacco exposure in utero, the ones with exposure excluded (n=144) and children with neonatal intensive care unit (NICU) stay excluded (n=132). Cluster color indicates significance as a z-value. The position of the green crosshair indicates the most statistically significant vertex in statistically significant clusters. Color coding of regions according to the Desikan-Killiany atlas. No correction for multiple comparisons was made.


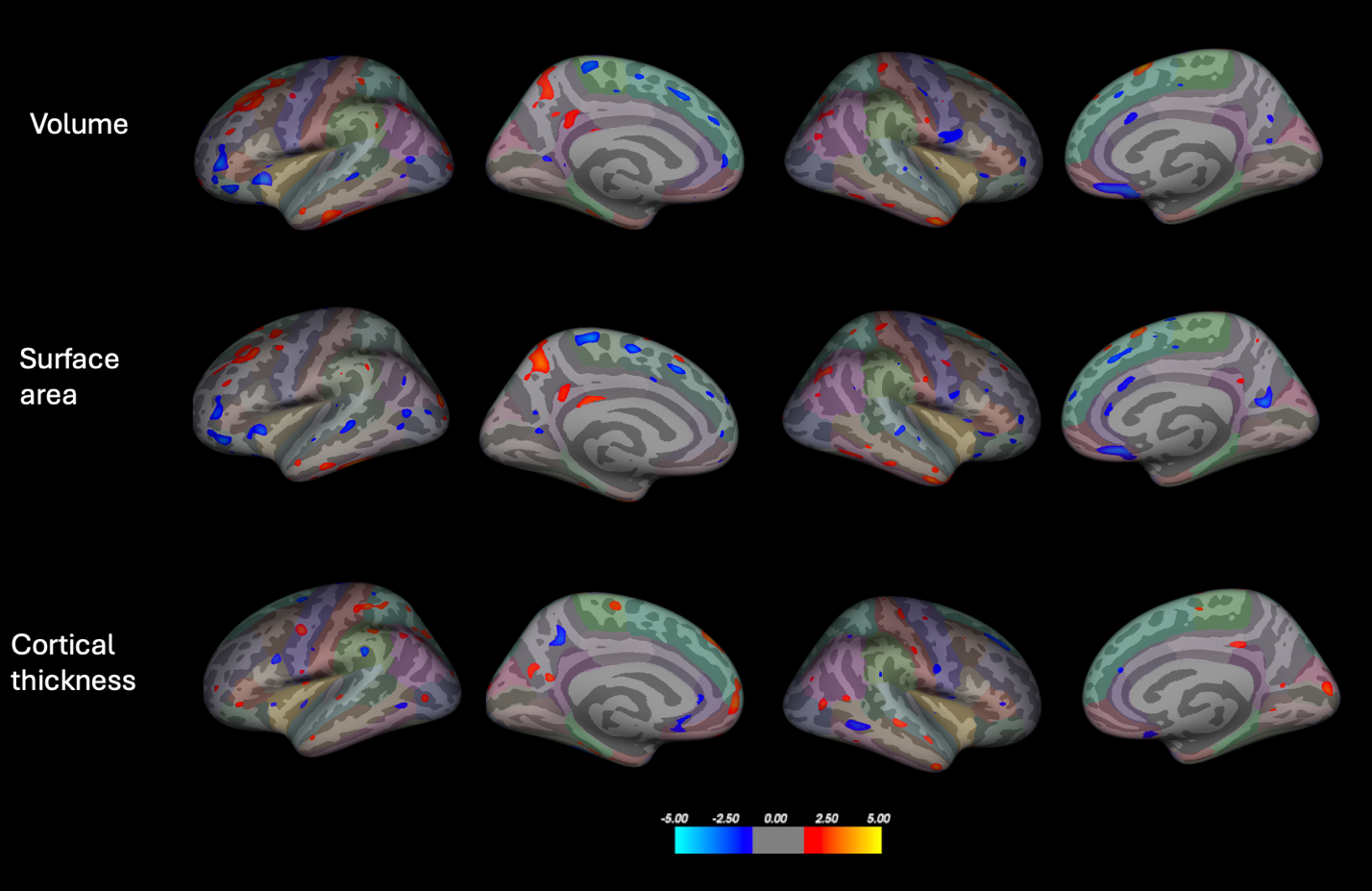


Supplementary Figure 4: The correlation between the Delay of Gratification -task and cortical volume presented on the top row, surface area in the middle and cortical thickness on the bottom row (n=165). The results were corrected for child’s gender, age at scan, ponderal index (mass in kilograms divided by height in meters cubed; measured during the neuroimaging visit), maternal age at term and maternal education level. There were no results found when the unreliable data was removed. There were 11 cases of unreliable data due to following 4 reasons: 1) testing related e.g. researcher error, incorrect task presentation, 2) child related e.g. restlessness, inability to follow orders, 3) parent present, 4) other. Color coding of regions according to the Desikan-Killiany atlas. No correction for multiple comparisons was made.
