# Supplementary material for: Effortful Control and Cortical Brain Structure in 5‐Year‐Old Children: Findings From the FinnBrain Birth Cohort Study
Source: Eur J Neurosci. 2026 Jun 10;63(11):e70580. doi: 10.1111/ejn.70580 (PMC13250741; doi:10.1111/ejn.70580)
Supplement: Supplementary file 2 — Data S1: We conducted a Pubmed search on 21 January 2024 with the following query: mri AND (structur* OR volume OR area OR ‘cortical thickness’) AND (infan* OR toddler OR child OR childre* OR adolesc*) AND (self‐control OR ‘effortful control’ OR ‘executive function’ OR ‘inhibitory control’) and the last two articles on the table are added from our previous searches. [file EJN-63-0-s001.docx]

**Supplementary file 1**

| **Study** | **Subjects Age** | **MRI** | **Neuropsychological Assessment Questionnaire** | **Covariates** | **Results** |
| --- | --- | --- | --- | --- | --- |
| (Feng et al., 2023) | N = 254 (152 with ADHD and 102 healthy controls) 6–17 years | sMRI and fMRI, 3T | BRIEF | age, sex | Only fMRI associations with BRIEF: IC_ref_fractional amplitude of low frequency fluctuation was significantly correlated with Behaviour regulation index and three of its factors (inhibition, shift and emotional control). This correlation was seen only on healthy controls whereas there was no correlation in ADHD patients |
| (Hadaya et al., 2023) | N = 251 MRI at 38-53 weeks post-menstrual age and neuropsychological assessments at 4–7 years | sMRI, fMRI and DWI, 3T | CBQ, SDQ, BRIEF–P | age, sex, multiple pregnancy | The subgroup that had highest EC scores also displayed larger relative volumes in the left insula and bilateral orbitofrontal cortex and higher degree centrality in an overlapping region in the left orbitofrontal cortex. |
| (Borges et al., 2023) | N = 677 aged 6 –14 years and N = 447 follow up after 3 years at 9 –17 years | sMRI, 1.5T | CBCL | age, sex | Association found with CBCL: Cerebellar volumes higher than expected for age and sex were associated with lower externalizing specific factor and higher executive functions. |
| (Hai et al., 2022) | N = 50 (26 with ADHD and 24 healthy controls) 7–16 | sMRI, 3T | BRIEF–2 | sex, ADHD | Negative association between cognitive regulation and right superior frontal gyrus cortical thickness. |
| (Raja et al., 2021) | N =71 7.5–8.5 years | sMRI, 1.5T | BRIEF | age, sex | No associations between MRI and the BRIEF areas of inhibition or emotional control. Structural lateralization index of total surface area of Insula positively correlated with Executive function domain related BRIEF scores (Metacognition and Working Memory). Structural lateralization index of total surface area of precuneus negatively correlated with executive function domain related BRIEF scores (Global Executive Composite, Metacognition and Plan/Organize). |
| (Badaly et al., 2022a) | N = 150 (55 with congenital heart disease and 95 healthy controls) 6–25 years | sMRI, 3T | BRIEF | age, intellectual functioning | No associations between MRI and the BRIEF areas of inhibition or emotional control. Positive association was found between working memory (BRIEF) and cerebellar, lateral prefrontal and medial prefrontal volumes. In addition, a positive association was found between planning/organization (BRIEF) and cerebellar volume. |
| (Rakesh et al., 2021) | N = 177 MRI at three age points at 13(T1), 17(T2), and 19(T3) years (N = 73 had 3 scans) | sMRI, 3T (multiple scanners) | EATQ–R (T1) | sex | A significant effect of EC on brainAGE (the difference between brain-predicted-age and chronological age investigated by using linear mixed models) trajectory found at T2, but not T1 or T3. The greatest reduction in t–statistic values was observed when excluding the frontal lobe and the cingulate region. |
| (T. Li et al., 2020) | N = 68, 8 years (90 –101 months) | sMRI, 1.5T | BRIEF | sex, age | No associations between MRI and the BRIEF areas of inhibition or emotional control. BRIEF (working memory) showed negative correlations with cortical GM volume in the left medial OFC and left pars opecularis gyrus. BRIEF (initiate) showed negative correlations with GM thickness in the right parahippocampal gyrus. |
| (Frere et al., 2020) | N = 335, testing at two age points: 14 and at 16 years | sMRI and DWI, 3T | The youth strengths inventory (self-report), SDQ | Total intracranial volume, sex | Sex differences in brain maturation on adolescents: amygdala and hippocampal volume increase in boys and decrease in girls between the testing points at age 14 and 16 years. These volumes had positive associations with emotional regulation: boys showed an increase on positive attributes, such as generosity and solicitude, whereas girls showed a decrease in these characteristics. |
| (Barch et al., 2019) | N = 306 age 3.0–5.11 years (T1) and N = 172 at age point 13.3–19.4 years (T9) | sMRI and fMRI, 3T | Emotion regulation checklist, caregiver assessment (T6–T9), Cognitive emotion regulation questionnaire, self-report (T6–T9) | age, sex, family income. Supplementary analyses controlling for current psychotropic medication and history of psychotropic medications. | Modest relationships of depression to hippocampal volume and strong relationships between emotion regulation and both episodic memory and hippocampal volume were found. These data are consistent with prior work in adults linking depression, episodic memory, emotion regulation, life stress/adversity, and hippocampal volume in adults and suggest similar relations are evident as early as adolescence when memory systems are under development. |
| (Cheetham et al., 2017) | N = 107 Age points: 12 (MRI anf EC assessment), 15 (SUD assessment), 16 (SUD) and 18 (SUD) | sMRI, 3T | EATQ-R | sex | Volumes in the left OFC and left lateral subregions were positively correlated with EC. Left OFC was seen to be the mediating factor between EC and SUD. |
| (Schlotz et al., 2014) | N = 27, 15–16 years | sMRI, 3T | EATQ-R | maternal smoking, drinking alcohol, parity, social class | Lower birth weight associated with lower inhibitory control and smaller surface area in the left and medial OFC and in the right inferior frontal gyrus, lower caudate volume and thicker medial OFC. In addition, an indirect effect of birth weight on inhibitory control via caudate volume was found. |
| (Blanton et al., 2010) | N = 72, 8–18 years | sMRI, 1.5T (multiple scanners) | BRIEF | Total cranial volume | In girls a smaller left amygdala volume was linked to better emotional control while in boys the finding was opposite, and a larger left amygdala volumes were linked to better emotional control. |
| (Fung et al., 2023) | T1 N=10574, 9–11 years and T2 N=9273, 11–14 years | sMRI, fMRI and DWI, 3T (The ABCD study http://abcdstudy.org 22 different research sites) | CBCL | age, sex, highest parental education, household income, and race, for neuroimaging also total intracranial volume | Adherence to sleep and screen time recommendations was associated with less behavioral problems and larger gray matter volumes. Associations between psychosocial health and brain structure not explored. |
| (Yu Chan et al., 2022) | MRI N=202 at 4,5 years and BRIEF-2 N=154 at 7–8,5 years | fMRI and DWI, 3T | BRIEF-2 | sex | No associations between structural and functional connectivity measures in either hemisphere and executive functionin measured by BRIEF-2. |
| (Vanes et al., 2021) | mri N=284 at term with median post-menstrual age of 42.57 [37.86–44.86] weeks, Questionnaires at preschool age n = 206; median age 4.65 [4.19-7.17] years | sMRI, 3T | CBQ very short form, SDQ, BRIEF | Gestational age, sex, post-menstrual age at scan, Index of Multiple Deprivation | Principal component analysis identified 3 PC's: PC1 (preterm phenotype), PC2 (cognitive) and PC3 (socio-emotional), of which PC1 and PC3 are closest to our study. PC1 was driven by positive findings in SDQ (ADHD symptoms), BRIEF (executive deficits) and social responsiveness scale (autism spectrum symptoms). Structural covariate networks in the fronto-insular area had a negative association with PC1. PC3 was driven by positive loadings of scales in the CBQ, Empathy questionnaire and SDQ. PC3 had no neuroanatomical associations. |
| (Owens et al., 2021) | N = 11875, 9–10 years | sMRI, fMRI and DWI, 3T (The ABCD study http://abcdstudy.org 22 different research sites) | CBCL | age, pubertal status, sex, handedness, internalizing symptoms, parental education, child race, parental income, medication | ADHD symptomatology was predicted by lower cortical surface area in the dorsolateral PFC, ventrolateral PFC, ACC, insula, lateral temporal cortex and lateral occipital cortex and lower cotical thickness in the ACC, left insula, medial temporal cortex, precentral gyrus and postcentral sulcus, and greater cortical thickness in the ventrolateral PFC, right insula and lateral occipital cortex. |
| (Thijssen et al., 2015) | N = 464, 6–9 years | sMRI, 3T | SDQ | age, sex, ethnicity, family income, maternal education | Prosocial behaviour is related to thicker cortex in a cluster found in a part of the left superior frontal and rostral middle frontal cortex. Gender moderated the association in a cluster including the right rostral middle frontal and superior frontal cortex as well as in a cluster covering the right superior parietal cortex, cuneus, and precuneus. |
| (Whittle et al., 2008) | N= 153, 11.4–13.7 years (mean 12.6 years) | sMRI, 3T | EATQ-R | whole brain volume, sex | Higher EC was linked to larger volume of the left orbitofrontal cortex and hippocampus. In addition, higher negative affectivity was linked to smaller volume of the left dorsal paralimbic and higher affiliativeness was associated with larger volume of the right rostal/ventral limbic portion of the ACC. Affiliativeness and surgency in females was linked to rostal/ventral ACC. |
| (Wang et al., 2019) | N = 148, 17–20 (mean age 18.51, SD 0.55) | sMRI, 3T | NEO Five-Factor Inventory, Chinese National University Entrance Exam, Raven's Advanced Progressive Matrices | general intelligence, family sosioeconomic status, "Big five" personality traits (other than conscientiousness) | Trait conscientiousness was positively associated with the GM volume in the bilateral superior parietal lobe and was negatively associated with the GM volume in the right middle fromtal gyrus. In addition, trait conscientious was found to be the mediating factor between academic performance and the brain regions mentioned above. |

*Supplementary file 1: We conducted a Pubmed search on January 21^st^ 2024 with the following query: mri AND (structur* OR volume OR area OR "cortical thickness") AND (infan* OR toddler OR child OR childre* OR adolesc*) AND (self-control OR "effortful control" OR "executive function" OR "inhibitory control") and the last two articles on the table are added from our previous searches.*

*BRIEF = Behaviour Rating Inventory of Executive Function (parental asessment)*

*ADHD = Attention Deficit Hyperactivity Disorder*

*CBQ = Child Behavioral Questionnaire*

*SDQ = Strengths and Difficulties Questionnaire (self-report)*

*CBCL = Child Behaviour Checklist*

*EATQ-R = Early Adolescent Temperament Questionnaire-Revised*

*SUD = Substance use disorder*

*PFC = Prefrontal cortex*

*ACC = Anterior cingulate cortex*

*GM = Grey matter*

SD = Standard deviation
